# High-Rate Continuous-Variable Quantum Key Distribution with Orbital Angular Momentum Multiplexing

**DOI:** 10.3390/e23091187

**Published:** 2021-09-09

**Authors:** Xinchao Ruan, Wenhao Shi, Guojun Chen, Wei Zhao, Hang Zhang, Ying Guo

**Affiliations:** 1 School of Automation, Central South University, Changsha 410083, China; rxc1126@csu.edu.cn; 2 School of Computer Science and Engineering, Changchun University of Technology, Changchun 130012, China; 204214@csu.edu.cn; 3 Jiangsu Key Construction Laboratory of IoT Application Technology, Taihu University, Wuxi 214064, China; 4 School of Computer Science and Engineering, Central South University, Changsha 410083, China; zwzhaowei@csu.edu.cn

**Keywords:** quantum key distribution, continuous-variable, orbital angular momentum, multiplexing

## Abstract

The secret key rate is one of the main obstacles to the practical application of continuous-variable quantum key distribution (CVQKD). In this paper, we propose a multiplexing scheme to increase the secret key rate of the CVQKD system with orbital angular momentum (OAM). The propagation characteristics of a typical vortex beam, involving the Laguerre–Gaussian (LG) beam, are analyzed in an atmospheric channel for the Kolmogorov turbulence model. Discrete modulation is utilized to extend the maximal transmission distance. We show the effect of the transmittance of the beam over the turbulent channel on the secret key rate and the transmission distance. Numerical simulations indicate that the OAM multiplexing scheme can improve the performance of the CVQKD system and hence has potential use for practical high-rate quantum communications.

## 1. Introduction

Continuous-variable quantum key distribution (CVQKD) has been developed in recent years and benefits from its high key generation rate and good compatibility with existing optical fiber communication systems [1,2,3]. To date, numerous sub-protocols have been derived from the GG02 protocol [4], and their security has been theoretically demonstrated [5,6]. In addition, experimental investigations have also demonstrated the feasibility of the designed scheme [7,8,9]. However, there are still obstacles that restrict the implementation of the practical CVQKD system [10,11]. For example, the transmission distance is limited, and the secret key rate is not high enough, hindering the practical application of the CVQKD system.

To overcome the obstacles mentioned above, several schemes have been proposed. For example, in order to extend the secure transmission distance of the key, non-Gaussian operation techniques, including photon addition and subtraction [12,13], quantum scissors [14,15,16], quantum catalysis [17,18,19], amplifiers [20,21,22], etc., have been suggested. Besides, It has been shown that discrete modulation has greater potential to realize long-distance transmission than Gaussian modulation [23,24]. On the other hand, multiplexing technology, such as wavelength division multiplexing [25], frequency division multiplexing [26], and polarization multiplexing [27], can be used to achieve high-rate communication. Furthermore, multiple multiplexing schemes can be utilized simultaneously in a communication system to further improve the channel capacity and spectrum efficiency [28,29]. Apart from the above-mentioned multiplexing schemes, orbital angular momentum (OAM) multiplexing is a novel multiplexing technology with low complexity and high spectral efficiency for short-distance communication [30,31]. It has attracted much attention in recent years because it can satisfy the enormous demands of communication systems for enhancing the information capacity [32,33,34].

As a new additional degree of freedom, OAM is completely independent from wavelength, frequency, polarization, and so on [35]. Unlike other degrees of freedom, the OAM of light can construct an infinite-dimensional Hilbert space, where the quantum coding can be achieved on the orthonormal basis formed by the OAM states [34,36]. Besides, due to the orthogonality between modes, the OAM beam can be used as an information carrier for efficient multiplexing and demultiplexing. It has infinite dimensional eigenstates in theory, which corresponds to infinite multiplexing channels when it is applied to a multiplexing scheme, thus achieving a higher key rate than other multiplexing schemes. Moreover, OAM multiplexing is compatible with other existing multiplexing techniques, and the simultaneous use of multiple multiplexing techniques can greatly improve channel capacity and spectrum efficiency to meet the bandwidth requirements of future high-capacity communications.

In this paper, we take the above two obstacles into account and propose an OAM multiplexing scheme to increase the secret key rate of the CVQKD system. The scheme is designed and the implementation process is described. We analyze the optical propagation characteristics of the Laguerre–Gaussian (LG) beam in an atmospheric turbulence channel and discuss the effect of crosstalk on OAM modes. The probability of different modes detected by the receiver is derived, which can be used to characterize the effective transmittance of the single-mode state in the turbulent channel. We find that the effective transmittance is negatively correlated with the intensity of atmospheric turbulence, the transmission distance, and the angular mode number of OAM. We calculate the secret key rate of the system in the asymptotic case and simulate the function relationship between the secret key rate and the transmission distance, the angular mode number, and the atmospheric refractive index structure constant. The simulation results show that in the CVQKD system based on OAM, the larger the angular mode number, the greater the influence of atmospheric turbulence on the mode, the lower the secret key rate, and the shorter the maximum transmission distance. In addition, by comparing the performance of the multiplexed and non-multiplexed schemes, we can find that OAM multiplexing can significantly improve the secret key rate of the system.

This paper is organized as follows. In Section 2, we suggest an OAM multiplexing scheme of CVQKD for performance improvement and analyze the propagation characteristics of the LG beam in turbulent atmospheric channels. In Section 3, the channel transmittance is calculated, the excess noise is analyzed, the secret key rate of the system is derived in the asymptotic scenario, and the system performance is evaluated from numerical simulations. Finally, the conclusion is drawn in Section 4.

## 2. CVQKD with OAM Multiplexing

In this section, we show an OAM multiplexing scheme for CVQKD and analyze the propagation characteristics of the LG beam in turbulent atmospheric channels.

### 2.1. OAM Multiplexing Scheme

**Figure 1:**
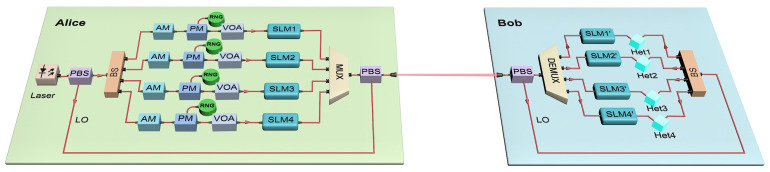
Schematic diagram of CVQKD system with OAM multiplexing. AM, amplitude modulator; PM, phase modulator; RNG, random number generator; VOA, variable optical attenuator; DEMUX, demultiplexing; MUX, multiplexing; SLM, spatial light modulator; PBS, polarization beam splitter; BS, beam splitters; Het, heterodyne detection.

As shown in Figure 1, we propose the schematic diagram of the OAM-based CVQKD protocol. It can be described as follows.

Step 1: A continuous-wave laser beam emitted by the transmitter (Alice) is divided into two parts after passing into a polarization beam splitter (PBS). One part is the weaker signal light, and the other is the stronger local oscillator (LO) light.

Step 2: The signal light is passed into a combination of beam splitters (BS) and is then equally divided into four parts. Each part is modulated by an amplitude modulator (AM) and a phase modulator (PM) to prepare four types of discrete states $|\alpha_{k}\rangle =|\alpha e^{i(2k+1)\pi /4}\rangle$ with modulation variance $V_{M}$, where k∈{0,1,2,3} and α satisfies $V_{M}=2\alpha^{2}$. The value of $V_{M}$ can be controlled by the AM. The modulated beams are respectively attenuated to a quantized level by a variable optical attenuator (VOA) and then passed into a spatial light modulator (SLM) loaded with different phase modes to prepare LG beams for OAM modes. The beams involving OAM modes are multiplexed together via a multiplexing module and then coupled with the LO light via a PBS.

Step 3: The coupled beam reaches the receiver (Bob) after transmission through a turbulent atmospheric channel with transmittance *T* and excess noise ε and then is separated into signal light and LO light by a PBS. The signal light is divided into four channels by a demultiplexing module, and each of them is demodulated by an SLM. It should be noted that the SLM here loads the spatial phase mode opposite to that of the SLM at the Alice side. Similarly, LO light is divided into four channels by a combination of BS and interferes with the demodulated signal light to perform coherence detection. The efficiency and electric noise of the imperfect detector can be denoted as η and $v_{el}$, respectively.

Step 4: Bob sends the measured results to Alice through the classical channel, and then the post-processing procedures, such as reverse reconciliation, privacy amplification, and so on, are carried out to establish the final secret key.

### 2.2. Propagation Characteristics of the LG Beam

Atmospheric turbulence (AT) is a factor that has an effect on the communication quality of the free-space CVQKD system. In a turbulent channel, the wavefront of the OAM beam is prone to distortion, which will lead to channel crosstalk and information mixing between the adjacent OAM modes. In this scheme, the LG beam is selected as the OAM beam, and its field distribution in cylindrical coordinates can be expressed as [37,38]

(1) $$\begin{array}{cc} u(r,\phi ,z)= & \sqrt{\frac{2p!}{\pi (p+|l|)!}}\frac{1}{\omega (z)}{\left[\frac{r\sqrt{2}}{\omega (z)}\right]}^{\left|l\right|}\times L_{p}^{\left|l\right|}\left[\frac{2r^{2}}{\omega^{2}\left(z\right)}\right]\times \exp\left[\frac{-r^{2}}{\omega^{2}\left(z\right)}\right]\times \\ & \exp\left[\frac{-ikr^{2}z}{2(z^{2}+z_{R}^{2})}\right]\exp[i(2p+|l|+1)\tan^{-1}\frac{z}{z_{R}}]\exp\left(il\phi \right), \end{array}$$

where *r* is the radial radius, ϕ is the azimuthal angle, *z* is the propagation distance, and *p* and *l* are the radial and angular mode numbers, respectively. The beam radius at distance *z* can be noted as $\omega \left(z\right)=\omega_{0}\sqrt{1+{\left(z/z_{R}\right)}^{2}}$, where $\omega_{0}$ is the radius of the zero-order Gaussian beam at the waist, $z_{R}=\pi \omega_{0}^{2}/\lambda$ is the Rayleigh range, and λ is the wavelength. $L_{p}^{l}\left(\cdot \right)$ represents the generalized Laguerre polynomial, and k=2π/λ is the wave number.

The turbulent channel can be simulated by inserting random phase screens at intervals of 50 m along the direction of propagation. Each screen is generated by shaping a set of complex Gaussian deviates according to a two-dimensional power spectrum of refractive index fluctuations [38]. The power spectrum considered here is the Wiener spectrum, and it can be expressed as

(2) $$\Phi \left(\kappa \right)=0.023r_{0}^{-5/3}{\left|\kappa \right|}^{-11/3},$$

where $\kappa \in [2\pi /L_{0},2\pi /l_{0}]$ is the spatial frequency, and $L_{0}$ and $l_{0}$ are the outer and inner scale of turbulence, respectively. $r_{0}=0.1853{\left(\frac{\lambda^{2}}{C_{n}^{2}z}\right)}^{3/5}$ is the atmospheric coherence length, and $C_{n}^{2}$ is the atmospheric refractive index structure parameter.

**Figure 2:**
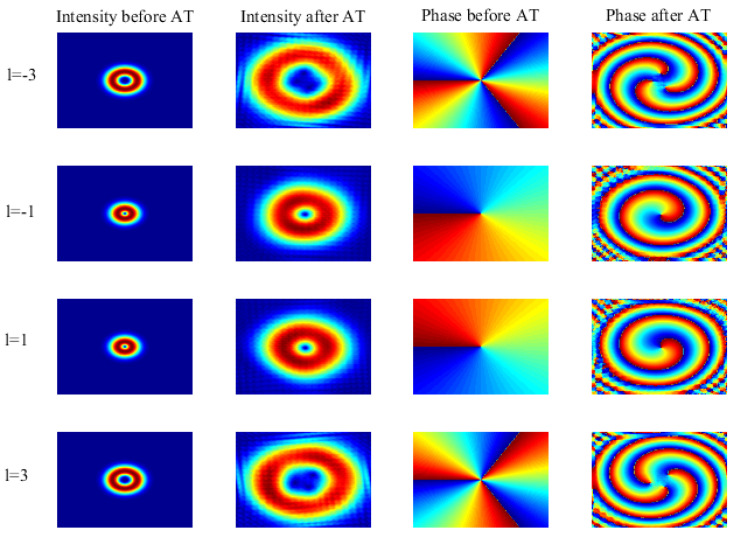
(Color online) Intensity and phase distribution of an LG beam before and after AT with OAM state l=−3,−1,1,3.

Figure 2 represents the intensity and phase patterns of the OAM beam with azimuth indices of l=−3,−1,1,3. Here, the phase screen distribution is equidistant with a total propagation of z=5 km and an adjacent phase screen interval of Δz=50 m. The numerical grid comprises 200×200 elements. The basic parameter settings are shown in Table 1. As can be seen from the figure, before being affected by AT, the spot size of the LG beam increases with the increase of the absolute value of the angular mode number *l*, and the intensity distribution of beams with the same absolute value of *l* is the same. The phase distribution is influenced by the sign of *l*; that is, the sign of *l* determines the direction of the helix phase gradient, and its absolute value determines the magnitude of the helix phase gradient. LG beams with the same absolute value but an opposite sign of *l* have opposite phase patterns. Under the influence of atmospheric turbulence, the intensity distribution of the LG beam tends to diverge, and its helical phase is also distorted, resulting in crosstalk between different modes. In addition, it can be seen from the figure that, under the same conditions, the influence of turbulence on the high-order vortex beam is greater than that on the low-order vortex beam.

The crosstalk between different modes caused by AT will cause the probability of the OAM mode being detected by the receiver to change; that is, the correct detection probability of the mode that should be detected is affected by other adjacent modes. For the Kolmogorov-based model, the probability of the crosstalk being detected by the receiver to different adjacent OAM states can be calculated by the following expression [39,40]

(3) $$P\left(l+\Delta l\right)=\frac{1}{\pi }\int_{0}^{1}\rho d\rho \int_{0}^{2\pi }\exp\left[-6.88\times 3^{\frac{2}{3}}{\left(\frac{r}{r_{0}}sin\frac{\Delta \theta }{2}\right)}^{5/3}\right]cos\left(\Delta l\Delta \theta \right)d\Delta \theta ,$$

where *l* is the initial OAM mode state, Δl is the difference between the detected OAM mode state and the initial mode state, ρ=r/R, *r* is the radial radius, *R* is the receiving aperture radius, $r_{0}$ is the atmospheric coherence length, and Δθ is the difference of azimuth coefficients before and after divergence.

**Figure 3:**
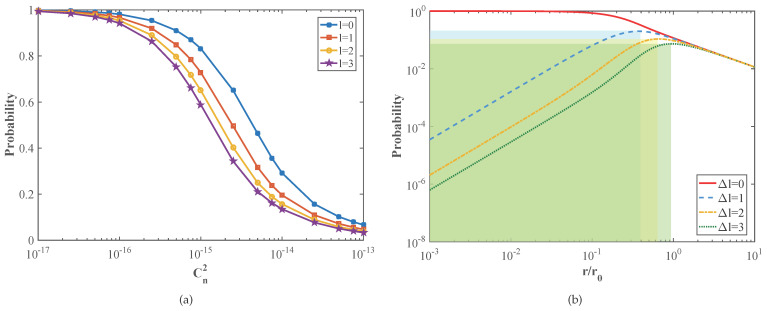
(**a**) Comparison of the probability of detecting the correct mode after AT. (**b**) Probability of detecting adjacent modes versus the ratio of radial coordinates to the atmospheric coherence length.

Here, we consider the influence of intermodal crosstalk caused by atmospheric turbulence on the system performance, while the loss of quantum state in the channel and receiver terminal is not taken into account. Therefore, we can simulate the influence of the atmospheric turbulence intensity on the probability of detecting the correct mode and the probability of cross-talk between modes, as shown in Figure 3. Figure 3a compares the probability of detecting the correct mode for different OAM modes after atmospheric turbulence. As can be seen from the figure, the larger the value of *l*, the lower the probability of detecting the correct mode, which shows that the influence of atmospheric turbulence mentioned above on the high-order vortex beam is greater than that on the low-order vortex beam. The influence of atmospheric turbulence on the cross-talk between OAM modes can be reflected by the probability of detecting other modes, as shown in Figure 3b. It should be noted that we only take the case of Δl=0,1,2,3 as examples here, where Δl=0 represents the case that the initial mode state is detected. The x-axis coordinate $r/r_{0}$ is the ratio of the radial coordinates to the atmospheric coherence length, which is positively correlated with the intensity of atmospheric turbulence. The shaded area represents the region where the probability of cross-talk increases with the enhancement of turbulence intensity. The simulation result shows that the initial OAM mode of the LG beam transmitted in a turbulent channel is scattered to the adjacent OAM modes, and the probability of the initial OAM mode being measured by the receiver decreases continuously. The probability of detecting adjacent OAM modes increases first and then decreases, and the more adjacent modes there are, the more likely they are to be detected. As the ratio $r/r_{0}$ increases—that is, the intensity of turbulence increases—the more adjacent modes there are, the more their detected probability will start to decay, which means that they will be more susceptible to the atmospheric turbulence.

## 3. Performance Analysis

In this section, we obtain the transmittance of the turbulent atmospheric channel, derive the secret key rate of the system, and evaluate the system performance.

### 3.1. Transmittance

When an LG beam propagates through an atmospheric channel, atmospheric turbulence will cause the refractive index to fluctuate, leading to a random phase distortion of the beam. Thus, the input state of OAM located in the finite dimensional space will be scattered to the adjacent mode, resulting in the error code of the CVQKD system. The intensity of atmospheric turbulence can be characterized by the refractive index structure constant $C_{n}^{2}$, and its classification criteria were proposed by Davis [41], as shown in Table 2. The effective transmittance of different OAM states propagating in the atmospheric channel can be characterized by the correct propagation probability of the initial mode detected by the receiver, namely [42]

(4) $$T\left(l,C_{n}^{2},z\right)=e^{-\mu z}\cdot P\left(l\right),$$

where μ is the link attenuation coefficient, *z* is the transmission distance, and P(l) is the probability of the initial mode being detected by the receiver. Further, the radial radius *r* and atmospheric coherence length $r_{0}$ in Equation (3) satisfy the following expression:

(5) $$\begin{array}{c} r=\sqrt{|l|+1}\cdot \omega \left(z\right),r_{0}=0.1853\cdot {\left(\frac{\lambda^{2}}{C_{n}^{2}z}\right)}^{3/5}. \end{array}$$

**Figure 4:**
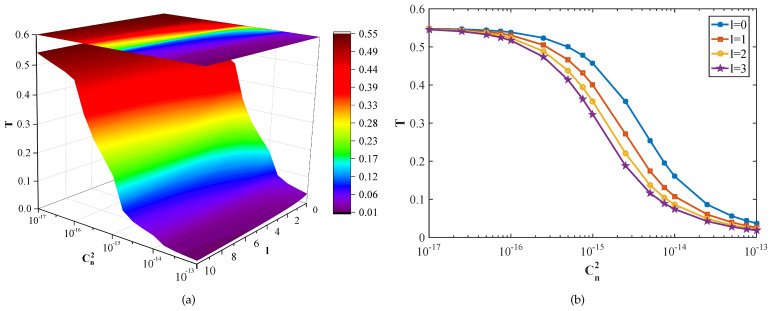
The effective transmittance *T* versus atmospheric refractive index structure constant $C_{n}^{2}$ and angular mode number *l*. (**a**) is the three-dimensional view. (**b**) represents a more intuitive plan.

**Figure 5:**
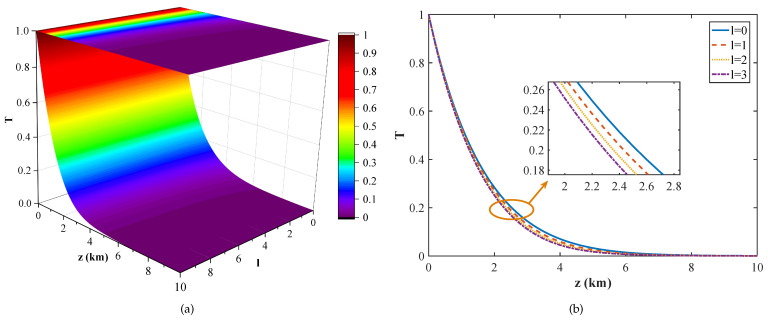
The effective transmittance *T* versus transmission distance *z* and angular mode number *l*. (**a**) is the three-dimensional view. (**b**) represents a more intuitive plan.

According to the above equations, the effective transmittance is mainly affected by the angular mode number *l*, the atmospheric refractive index structure constant $C_{n}^{2}$, and the transmission distance *z*. Figure 4 shows the effective transmittance of the turbulent atmospheric channel for the OAM-multiplexed CVQKD protocol with different turbulence intensities and angular mode numbers. Here, the transmission distance is set to 1 km. It can be seen from the figures that the effective transmittance decreases with the increase of turbulence intensity. When the turbulence intensity is the same, the smaller the angular mode number, the greater the transmittance. It can also be found from the Figure 4b that the effective transmittance is insensitive to the angular mode number in the case of strong turbulence and weak turbulence, while it is more sensitive to it in the case of moderate turbulence. It should be noted that, for the angular mode number *l* with the same absolute value, the effective transmittances are affected by atmospheric turbulence in the same way. Without loss of generality, we consider the case that the angular mode numbers are positive. In addition, the influence of transmission distance on the effective transmittance of the OAM-based CVQKD protocol is shown in Figure 5. Here, the atmospheric refractive index structure constant is set to $1\times 10^{-16}$ $m^{-2/3}$. We find that the effective transmittance decreases rapidly from the start and then becomes gradual with the increase of transmission distance. For the given transmission distance, the transmittance decreases with the increase of the angular mode number, as shown in Figure 5b.

### 3.2. Excess Noise

In the free space CVQKD with OAM multiplexing, there are many factors that may cause excess noise, such as imperfections of the modulator and detector, crosstalk between modes, phase perturbation, and so on. In the free space atmospheric channel, it is the atmospheric turbulence that causes the spiral spatial phase structure of the OAM beam to change, so that crosstalk occurs between different OAM modes, and as a result, an additional crosstalk noise is introduced. For the orthogonal field phase used for information encoding, atmospheric turbulence will also cause disturbance, which will also introduce an additional noise [43]. Fortunately, an existing study has shown that this phase disturbance can be eliminated by phase compensation [44]. Therefore, we mainly consider the noise introduced by mode crosstalk.

It can be seen from Section 2.2 that the OAM modes with the same absolute value are subject to the same interference effects of other modes, while the modes with different absolute values are subject to the different interference effects of other modes. This means that the modes with different absolute values are affected differently by crosstalk noises during transmission in the channel. Therefore, in the proposed multiplexing scheme, we need to independently analyze the excess noise in the sub-channels where the different modes are transmitted.

At the output of the channel, the excess noise for different mode can be expressed as

(6) $$\varepsilon_{out}=\varepsilon_{limit}+\frac{\tau P_{crosstalk}}{hv_{c}},$$

where $\varepsilon_{limit}$ represents the original excess noise of the CVQKD system, and we take its maximum value of 0.01 SNU (shot noise unit) here for simplicity. τ=1 ns is the effective sampling period, *h* is Planck’s constant, $v_{c}$ is the frequency of the noise photons, and $hv_{c}=1.28\times 10^{-19}$ J for a 1550 nm photon. $P_{crosstalk}$ is the noise power induced by crosstalk, and it can be expressed as

(7) $$P_{crosstalk}\left(l\right)=\sum_{i=1}^{3}P\left(l_{i}+\Delta l_{i}\right)\cdot P_{in},$$

where $P(l_{i}+\Delta l_{i})$ is the crosstalk probability of the other three modes to the mode *l*, which can be calculated by Equation (3), and $P_{in}=-85.9$ dBm is the input power of OAM light in each path. After the excess noise is normalized to the input of the channel, it can be noted as

(8) $$\varepsilon =\varepsilon_{out}/T,$$

where *T* is the channel transmittance, and its expression is shown in Equation (4).

**Figure 6:**
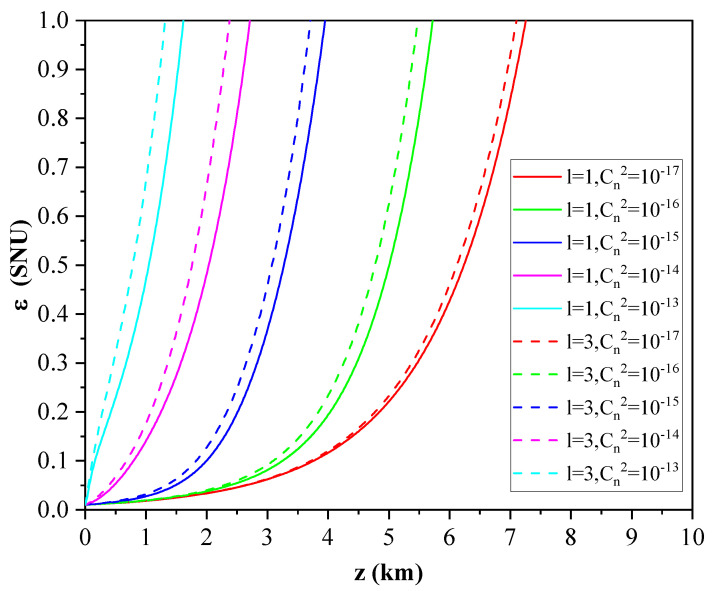
The function relationship between the excess noise and the transmission distance of the l=1 and l=3 modes under different turbulence intensities.

As shown in Figure 6, we have simulated the relationship between the excess noise and the transmission distance of the l = 1 and l = 3 modes under different turbulence intensities. It can be seen from the figure that the excess noise increases with the order of the OAM mode, the transmission distance, and the intensity of atmospheric turbulence. The greater the turbulence intensity, the more obviously the excess noise increases, which is the reason for the reduction of the secure communication distance. The higher the order of OAM, the stronger the interference from other modes, and the greater the mode crosstalk noise, which in turn will lead to the degradation of system performance.

### 3.3. Secret Key Rate

According to the above-mentioned analysis, we find that for OAM states with different angular mode numbers, the effective transmittance and excess noise in the turbulent atmosphere channel are different, and hence the key generation rate of the resulting CVQKD system is correspondingly different. In the proposed scheme, the multiplexing of four OAM modes with l∈{−3,−1,1,3} is taken into account. Therefore, the final secret key rate with reverse reconciliation in the asymptotic case can be expressed as

(9) $$K=\sum_{i=1}^{4}K_{i}=\sum_{i=1}^{4}\left[\beta I_{AB}\left(l_{i}\right)-\chi_{BE}\left(l_{i}\right)\right],$$

where *i* is the number of OAM modes multiplexed, β is the reconciliation efficiency, $I_{AB}$ is the Shannon mutual information between Alice and Bob, and $\chi_{BE}$ is the Holevo bound between Bob and Eve.

For heterodyne detection [45], we have

(10) $$I_{AB}=log_{2}\left(\frac{V+\chi_{tot}}{1+\chi_{tot}}\right),$$

where $V=V_{M}+1$, $\chi_{tot}=\chi_{line}+\chi_{het}/T$, $\chi_{line}=1/T+\varepsilon -1$, $\chi_{het}=\left(2+2v_{el}-\eta \right)/\eta$. Given that the value of the modulation variance we take is relatively small, the Holevo bound $\chi_{BE}$ can be calculated approximately, namely [23]

(11) $$\chi_{BE}=G\left(\frac{v_{1}-1}{2}\right)+G\left(\frac{v_{2}-1}{2}\right)-G\left(\frac{v_{3}-1}{2}\right)-G\left(\frac{v_{4}-1}{2}\right),$$

where

(12) $$\begin{array}{cc} G(x) & =\left(x+1\right)log_{2}\left(x+1\right)-xlog_{2}\left(x\right),\\ v_{1,2} & =\sqrt{\frac{1}{2}\left(A\pm \sqrt{A^{2}-4B}\right)},\\ v_{3,4} & =\sqrt{\frac{1}{2}\left(C\pm \sqrt{C^{2}-4D}\right)}, \end{array}$$

with

(13) $$\begin{array}{cc} A & =V^{2}+T^{2}{\left(V+\chi_{line}\right)}^{2}-2TZ_{4}^{2},\\ B & ={\left(TV^{2}+TV\chi_{line}-TZ_{4}^{2}\right)}^{2},\\ C & =\frac{A\chi_{het}^{2}+B+1+2\chi_{het}\left[V\sqrt{B}+T\left(V+\chi_{line}\right)\right]+2TZ_{4}^{2}}{{\left[T\left(V+\chi_{tot}\right)\right]}^{2}},\\ D & =\frac{{\left(V+\chi_{het}\sqrt{B}\right)}^{2}}{{\left[T\left(V+\chi_{tot}\right)\right]}^{2}}. \end{array}$$

Here we have

(14) $$\begin{array}{cc} Z_{4} & =2\alpha^{2}\sum_{k=0}^{3}\sqrt{\frac{\lambda_{k-1}^{3}}{\lambda_{k}}},\\ \lambda_{0,2} & =\frac{1}{2e^{\alpha^{2}}}\left[cosh\left(\alpha^{2}\right)\pm cos\left(\alpha^{2}\right)\right],\\ \lambda_{1,3} & =\frac{1}{2e^{\alpha^{2}}}\left[sinh\left(\alpha^{2}\right)\pm sin\left(\alpha^{2}\right)\right]. \end{array}$$

**Figure 7:**
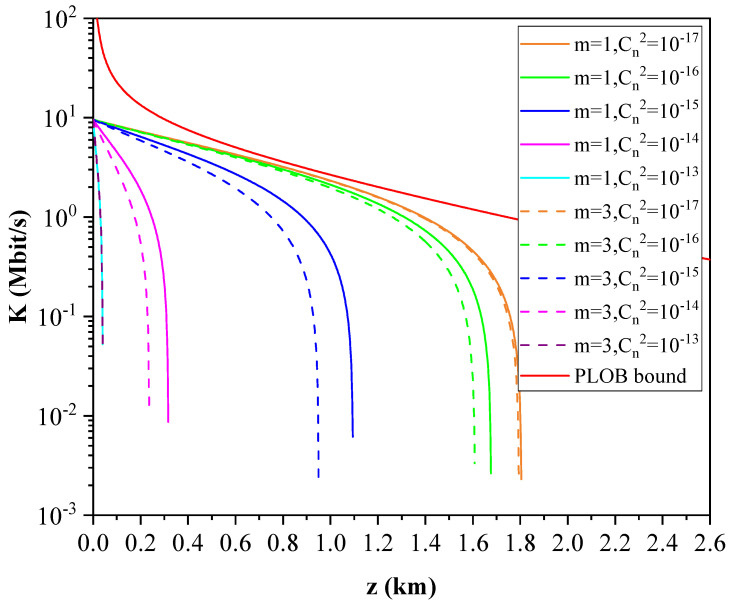
Secret key rates versus transmission distance for the OAM-multiplexed CVQKD protocol with different angular mode numbers and turbulent intensities. The basic parameters are set to $V_{M}=0.4,\;v_{el}=0.13,\;\eta =0.8,\;\beta =0.9$.

Based on the above analysis, the effects of atmospheric turbulence intensity and the angular mode number on the performance of the CVQKD system can be evaluated. We first consider the performance of the branches where different OAM modes are located in the multiplexing scheme. The system repetition rate is 100 MHz. As shown in Figure 7, the function relationships between the secret key rate and the transmission distance for the two modes of l=1 and l=3 under different turbulence intensities are obtained. We find that the secret key rate and the maximum transmission distance of the system decrease with the increase of turbulence intensity ($C_{n}^{2}=10^{-17}$ for weaker turbulence, $C_{n}^{2}=10^{-15}$ for medium turbulence, and $C_{n}^{2}=10^{-13}$ for stronger turbulence). Similarly, they all decrease with the increase of the angular mode number under the same turbulence intensity. In addition, the effect of atmospheric turbulence intensity on the system performance is more than that of the angular mode number of OAM.

**Figure 8:**
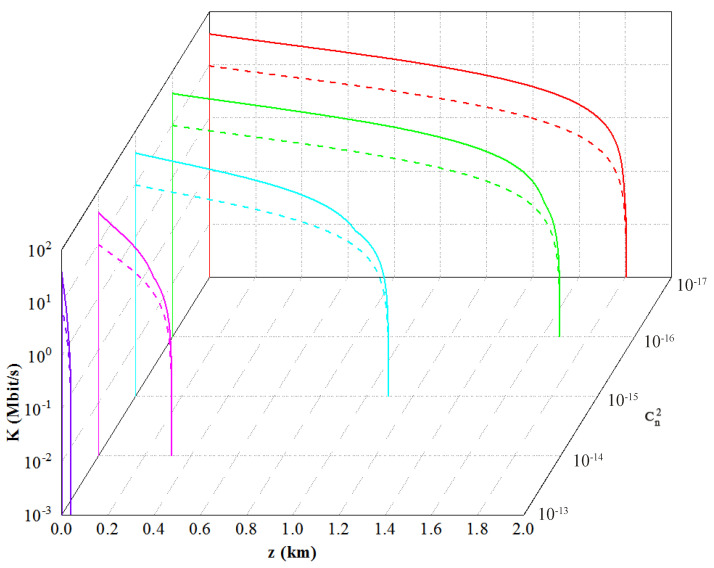
Secret key rates versus atmospheric refractive index structure constant and transmission distance for the OAM-multiplexed and single-mode CVQKD protocol. The solid line represents the multiplexing scheme, and the dashed line represents the single-mode case.

As shown in Figure 8, we simulate the relationship between the secret key rate *K*, atmospheric refraction index structure constant $C_{n}^{2}$, and transmission distance *z* for the OAM-multiplexed CVQKD protocol and compare it with that of a single mode in the multiplexing scheme. Here, we choose the case with the best performance—that is, the case of l=1—as a comparison. Simulation results show that the secret key rate of the system decreases with the increase of the transmission distance and the turbulence intensity, and the maximum secure transmission distance of the key gradually decreases with the increase of the turbulence intensity. The maximum secret key rate of the system can reach 38.31 Mbit/s in the multiplexed scheme, which is higher than that in the single-mode case. In contrast, the maximum transmission distance is the same as that of the latter with l=1.

## 4. Conclusions

We have suggested an OAM-based CVQKD protocol with discrete modulation over atmospheric turbulence channels to achieve high-rate quantum communications. The OAM multiplexing scheme is designed and the optical propagation characteristics of the LG beam are analyzed in this work. The influence of atmospheric turbulence on the crosstalk between OAM modes can be quantified by the probability of the initial mode being detected by the receiver. Consequently, the effective transmittance of different modes and excess noise in the turbulent atmosphere channel can be characterized. The simulation results show that the secret key rate of the CVQKD system decreases with the increase of transmission distance, atmospheric turbulence intensity, and angular mode number, and the key rate of the CVQKD system with multiple OAM modes of multiplexing can be greatly improved compared with that of a single mode. Therefore, we can further improve the secret key rate by increasing the number of multiplexed modes. This scheme is currently only suitable for short-distance transmission, and further work will focus on how to eliminate crosstalk between modes to further improve system performance.

## Figures and Tables

**Table 1:** Simulation parameters of LG beam.

Parameter	*p*	$\omega_{0}$/cm	$C_{n}^{2}/$ $m^{-2/3}$	λ/nm	$l_{0}/$m	$L_{0}/$m	z/km
Value	0	3	$1\times 10^{-14}$	1550	0.01	*∞*	5

**Table 2:** Division of atmospheric turbulence intensity.

Intensity	Weak Turbulence	Intermediate Turbulence	Strong Turbulence
$C_{n}^{2}\left(m^{-2/3}\right)$	<$6.4\times 10^{-17}$	$6.4\times 10^{-17}<C_{n}^{2}<2.5\times 10^{-13}$	>$2.5\times 10^{-13}$

## Data Availability

The data used are included in the article.
